# Multicentre cohort study of the impact of percutaneous coronary intervention on patients with concurrent cancer and ischaemic heart disease

**DOI:** 10.1186/s12872-021-01968-w

**Published:** 2021-04-13

**Authors:** Tatsuya Nishikawa, Toshitaka Morishima, Sumiyo Okawa, Yuki Fujii, Tomoyuki Otsuka, Toshihiro Kudo, Takeshi Fujita, Risa Kamada, Taku Yasui, Wataru Shioyama, Toru Oka, Takahiro Tabuchi, Masashi Fujita, Isao Miyashiro

**Affiliations:** 1 Department of Onco-Cardiology, Osaka International Cancer Institute, 3-1-69, Otemae, Chuo-ku, Osaka City, 541-8567 Japan; 2 Cancer Control Centre, Osaka International Cancer Institute, Osaka, Japan; 3 Department of Clinical Oncology, Osaka International Cancer Institute, Osaka, Japan

**Keywords:** Onco-cardiology, PCI, IHD, Colorectal, Lung, prostate, gastric, Prognosis, Oncology, Cardiology

## Abstract

**Background:**

The incidence of concurrent cancer and ischaemic heart disease (IHD) is increasing; however, the long-term patient prognoses remain unclear.

**Methods:**

Five-year all-cause mortality data pertaining to patients in the Osaka Cancer Registry, who were diagnosed with colorectal, lung, prostate, and gastric cancers between 2010 and 2015, were retrieved and analysed together with linked patient administrative data. Patient characteristics (cancer type, stage, and treatment; coronary risk factors; medications; and time from cancer diagnosis to index admission for percutaneous coronary intervention [PCI] or IHD diagnosis) were adjusted for propensity score matching. Three groups were identified: patients who underwent PCI within 3 years of cancer diagnosis (n = 564, PCI + group), patients diagnosed with IHD within 3 years of cancer diagnosis who did not undergo PCI (n = 3058, PCI-/IHD + group), and patients without IHD (n = 27,392, PCI-/IHD- group). Kaplan–Meier analysis was used for comparisons.

**Results:**

After propensity score matching, the PCI + group had better prognosis (n = 489 in both groups, hazard ratio 0.64, 95% confidence interval 0.51–0.81, *P* < 0.001) than the PCI-/IHD + group. PCI + patients (n = 282) had significantly higher mortality than those without IHD (n = 280 in each group, hazard ratio 2.88, 95% confidence interval 1.90–4.38, *P* < 0.001).

**Conclusions:**

PCI might improve the long-term prognosis in cancer patients with IHD. However, these patients could have significantly worse long-term prognosis than cancer patients without IHD. Since the present study has some limitations, further research will be needed on this important topic in cardio-oncology.

**Supplementary Information:**

The online version contains supplementary material available at 10.1186/s12872-021-01968-w.

## Background

Continued advances in cancer treatment have led to dramatic increases in the number of survivors [1]. As a result, the incidence of those suffering from concomitant coronary artery diseases (CAD) and cancer is also increasing. Some studies have reported that cancer itself, as well as cancer therapy, increases the risk of cardiovascular events [2–4]. However, cancer patients have historically been excluded from most CAD intervention trials. With the recent introduction of the field of onco-cardiology, patients suffering from both diseases simultaneously are attracting significant attention from oncologists and cardiologists [5, 6]. Several studies have shown that cancer patients undergoing percutaneous coronary intervention (PCI) exhibit higher all-cause mortality, bleeding, and other adverse cardiovascular events when compared with patients who have no history of cancer [7–13]. This raises the question of whether PCI can improve long-term prognosis in patients with cancer and comorbid ischaemic heart disease (IHD).

Including cancer patients in studies is challenging, given the wide heterogeneity in cancer type, stage, and treatment. Recently, Potts et al. [10] compared the short-term outcomes of PCI in prostate, breast, colorectal, and lung cancer patients with those of patients with a history of cancer and those with no cancer. They found that patients with metastatic disease had worse prognoses. They also noted that the rate of each adverse event varied by cancer type. However, data on the long-term prognosis of cancer patients undergoing PCI has not been reported in the literature. Thus, this report presents a comparison of all-cause mortality between cancer patients with IHD who underwent PCI and cancer patients with IHD who did not undergo PCI. Furthermore, this study aimed to determine how the long-term prognosis of cancer patients with IHD who underwent PCI differed from those who did not undergo PCI, as well as those without concurrent cancer and IHD.

## Methods

The study was approved by the local ethics committee of Osaka International Cancer Institute (Approval number: 1707105108) and the study protocol was in accordance with the principles set out in the 1964 Declaration of Helsinki. The requirement for informed consent was waived due to the retrospective nature of the study.

### Data sources

This was a multicentre retrospective cohort study using the Osaka Cancer Registry (OCR) and administrative data [14–18]. The OCR is a population-based cancer registry that compiles information on cancer diagnoses and outcomes in patients residing in Osaka Prefecture, Japan. OCR data include age, sex, history of smoking, type of cancer, date of cancer diagnosis, date of the last follow-up, date of any cause of death, and cancer stage (i.e., localised, regional to lymph nodes, regional by direct extension, and metastatic) according to SEER (surveillance, epidemiology, and end results) [19]). The OCR also includes treatment information (i.e., curative surgery/endoscopic treatment, chemotherapy, hormonal therapy, and radiation therapy). Cancer types are defined according to the International Classification of Diseases for Oncology, Third Edition (ICD-O-3). Furthermore, administrative data from Japan’s Diagnosis Procedure Combination Per-diem Payment System (DPC) were collected from 36 designated cancer care hospitals in Osaka Prefecture. The DPC data include medication and history of PCI. In addition, upon hospital admission, patient data on activities of daily living (ADL; Barthel Index score), smoking habits, and International Classification of Diseases, Tenth Revision (ICD-10) diagnoses are recorded. OCR data are linked to administrative data at the patient level, using each hospital’s patient identification number.

### Study population

**Fig. 1:**
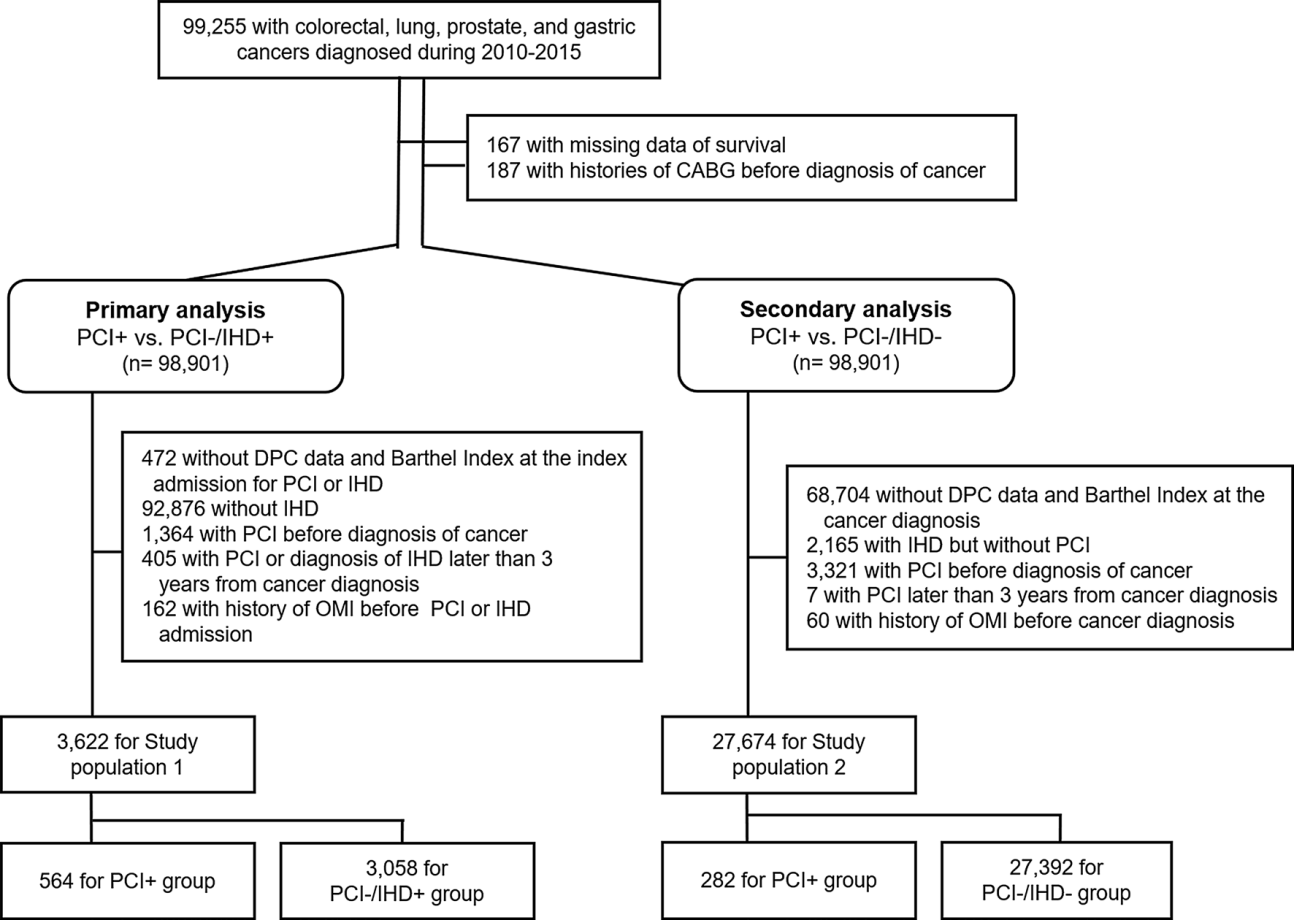
Study flowchart. *CABG* coronary artery bypass grafting, *PCI* percutaneous coronary intervention, *IHD* ischaemic heart disease, *DPC* Japan’s Diagnosis Procedure Combination Per-diem Payment System, *OMI* old myocardial infarction

Study investigators identified gastric (ICD-O-3 topographical codes: C16.x), colorectal (C18.x-C20.x), prostate (C61.x), and lung (C34.x) cancer patients who were diagnosed between 2010 and 2015. This decision was based on data that patients with these cancers underwent PCI most frequently (see Additional file 1: Figure S1). Exclusion criteria included a number of items: having undergone coronary artery bypass grafting (CABG), history of myocardial infarction, history of PCI, and missing data (including vital status, DPC, and/or other baseline characteristics) at index admission for PCI or IHD for primary analysis (described below) and at cancer diagnosis for secondary analysis (described further on). The patient selection flowchart can be seen in Fig. 1. The presence of IHD, including angina pectoris, asymptomatic myocardial ischemia, and acute myocardial infarction, was determined as a patient receiving IHD as the main diagnosis, having IHD as comorbidity upon admission, or having IHD as an in-hospital complication of index admission based on ICD-10 in DPC data (see Additional file 1: Table S1).

### Exposure

Patients were categorised into 3 groups: (1) those diagnosed with IHD who underwent PCI (the PCI + group); (2) those diagnosed with IHD who did not undergo PCI (the PCI-/IHD + group); and (3) those without a diagnosis of IHD (PCI-/IHD- group). To assess its effects on long-term prognosis, only patients who underwent PCI within 3 years of their cancer diagnosis were included in the PCI + group. The 3-year threshold was chosen because it includes 90% of patients undergoing PCI after the diagnosis of cancer. Among patients with IHD not undergoing PCI, only those who had been diagnosed with IHD within 3 years of their cancer diagnosis were included in the PCI-/IHD + group. As a sensitivity analysis, all-cause mortality was also assessed for patients who had undergone PCI or had received a diagnosis of IHD within 1.5 years of their cancer diagnosis.

### Potential confounders

Data on medications (statins, β-blockers, angiotensin-converting enzyme [ACE] inhibitors, angiotensin II receptor blockers, and oral anticoagulants [warfarin and direct oral anticoagulants]), coronary risk factors (hypertension, dyslipidaemia, diabetes mellitus, and overweight); and other confounders (atrial fibrillation, congestive heart failure, and chronic kidney disease) were retrieved from the DPC database according to ICD-10 codes (see Additional file 1: Table S1). The medication was considered in the analysis if it had been introduced before discharge from the index hospitalisation (see Additional file 1: Table S2). Overweight status was defined as a body mass index > 25 kg/m^2^. The Barthel Index was used to measure ADL, and patients were divided into 3 groups based on their scores: 0–39, 40–59, and 60–100.

### Statistical analysis

In the primary analysis, we analysed the effect of PCI on long-term all-cause mortality in cancer patients with IHD by comparing the PCI + and PCI-/IHD + groups. Survival was calculated from the index admission for PCI or IHD. Subgroup analysis by cancer type was also performed. In the secondary analysis, the PCI+ and PCI−/IHD− groups were compared to examine the combined impact of PCI and IHD on cancer prognosis. Survival was calculated from the index cancer diagnosis. As a sensitivity analysis, the difference in all-cause mortality between the PCI + group, excluding those with the acute coronary syndrome (ACS), and the PCI−/IHD− group was assessed.

Propensity score-matched survival analyses were performed in both primary and secondary analyses. The propensity score for PCI treatment was calculated using all 22 covariates described in Table 1. For the subgroup analysis of lung cancer patients, small cell carcinoma (ICD-O-3 morphological codes: 8041-8045) was also included as a factor. After 1:1 matching, 5-year all-cause mortality was assessed using Kaplan–Meier analysis. Caliper width was set as 0.2 times the standard deviation of the propensity scores. The balance of each factor was assessed using the standardised difference. Since the time interval between cancer diagnosis and admission for PCI varied, it was considered to represent an immortal time bias in the secondary analysis. Consequently, we used extended Kaplan–Meier analysis by adjusting for immortal time bias [20–22] after propensity score matching. In the PCI + group, the number at risk during the interval between cancer diagnosis and admission for PCI was 0. Therefore, PCI + group patients were grouped with PCI−/IHD− group patients during no-risk periods, with survival analysis in both groups starting at the date of cancer diagnosis [22].

**Table 1:** Baseline characteristics of the PCI + and PCI−/IHD− groups for the primary analysis

	All patients (n = 3622)		Entire cohort					Propensity score-matched sample				
			PCI+ (n = 564)		PCI−/IHD+ (n = 3058)		SD^*^	PCI+ (n = 489)		PCI−/IHD+ (n = 489)		SD^*^
Age, mean ± standard deviance	74	± 7.8	72	± 7.1	74	± 7.9	0.209	73	± 7.1	73	± 7.7	0.015
Sex							0.231					0.035
Female	785	(22)	80	(14)	705	(23)		74	(15)	68	(14)	
Male	2837	(78)	484	(86)	2353	(77)		415	(85)	421	(86)	
Cancer type												
Colorectal cancer	1165	(32)	195	(35)	970	(32)	0.061	174	(36)	170	(35)	0.005
Lung cancer	910	(25)	115	(20)	795	(26)	0.133	103	(21)	104	(21)	0.005
Prostate cancer	505	(14)	124	(22)	381	(12)	0.254	97	(20)	98	(20)	0.010
Gastric cancer	1042	(29)	130	(23)	912	(30)	0.154	115	(23)	117	(24)	0.080
Cancer stage												
In situ	252	(7)	48	(9)	204	(7)	0.070	45	(9)	41	(8)	0.016
Localised	1836	(51)	297	(53)	1539	(50)	0.047	253	(52)	249	(51)	0.030
Regional to lymph nodes involved	471	(13)	68	(12)	403	(13)	0.034	61	(12)	66	(14)	0.052
Regional by direct extension	399	(11)	78	(14)	321	(11)	0.102	66	(14)	75	(15)	0.050
Distant site(s)/node(s) involved	579	(16)	53	(9)	526	(17)	0.231	48	(10)	41	(8)	0.011
Unknown	85	(2)	20	(3)	65	(2)	0.086	16	(3)	17	(4)	0.056
Barthel index score												
60–100	3206	(89)	489	(87)	2717	(89)	0.066	428	(87)	427	(87)	0.006
40–59	153	(4)	21	(3)	132	(4)	0.030	19	(4)	15	(3)	0.045
0–39	263	(7)	54	(10)	209	(7)	0.100	42	(9)	47	(10)	0.035
Overweight	968	(27)	164	(29)	804	(27)	0.069	144	(29)	148	(30)	0.018
Current or past smoking	1986	(55)	328	(58)	1685	(54)	0.090	283	(58)	277	(57)	0.025
Dyslipidemia	1052	(29)	323	(57)	729	(24)	0.740	259	(53)	252	(52)	0.029
Hypertension	1851	(51)	384	(69)	1467	(48)	0.417	320	(65)	319	(65)	0.004
Diabetes mellitus	1179	(33)	261	(46)	918	(30)	0.319	217	(44)	219	(45)	0.008
Chronic kidney disease	252	(7)	47	(9)	205	(7)	0.068	44	(9)	49	(10)	0.035
Congestive heart failure	635	(18)	168	(30)	467	(15)	0.343	133	(27)	148	(30)	0.068
Atrial fibrillation	309	(9)	63	(11)	246	(8)	0.091	52	(11)	47	(10)	0.034
β-blocker	2773	(77)	230	(41)	619	(20)	0.476	185	(38)	187	(38)	0.008
Statin	2499	(69)	306	(54)	817	(27)	0.610	255	(52)	259	(53)	0.016
ACE inhibitor	296	(8)	90	(16)	206	(7)	0.303	68	(14)	71	(14)	0.012
ARB	1472	(41)	204	(36)	959	(31)	0.118	182	(37)	191	(39)	0.038
Oral anti-coagulants	447	(12)	68	(12)	379	(12)	0.002	63	(13)	64	(13)	0.006
Acute coronary syndrome	650	(18)	185	(33)	465	(15)	0.416	134	(27)	129	(26)	0.023
Days from cancer diagnosis to PCI/IHD admission, median (IQR)	78	(31–384)	274	(98–607)	65	(27–325)	0.555	250	(85–588)	279	(48–599)	0.015
Chemo/radiation/hormonal therapy	1242	(34)	168	(30)	1074	(35)	0.119	143	(29)	156	(32)	0.058
Surgery or endoscopic resection	2300	(64)	369	(65)	1931	(63)	0.058	323	(66)	322	(66)	0.004

Data are presented as n (%) unless otherwise indicated

*ACE* angiotensin-converting enzyme, *ARB* angiotensin II receptor blocker, *IHD* ischaemic heart disease, *IQR* interquartile range, *PCI* percutaneous coronary intervention, *SD* standardised difference, *PCI+* cancer patients undergoing PCI, *PCI−/IHD* cancer patients without IHD and not undergoing PCI

Cox proportional hazard analysis with inverse probability of treatment weighting (IPTW) for 5 years from PCI or IHD admission was also performed to confirm the robustness of the results. The entire cohort was weighted by stabilised average treatment effect weight [23]. Proportional hazards assumptions were confirmed by Schoenfeld residuals. For further confirmation, multivariable Cox proportional hazard analysis of the propensity score-matched sample was performed with a history of PCI, age (continuous variable), sex, cancer type, cancer stage, Barthel Index, ACS, and interval from cancer diagnosis to index admission for PCI or IHD as covariates for the primary analysis. Each of these variables, except ACS and interval from cancer diagnosis to index admission for PCI or IHD, was used for the secondary analysis.

JMP (version 11.0; SAS Inc., Tokyo, Japan) was used for data organisation and propensity score matching while graphing and all other analyses were performed using STATA (version 15; STATA Corporation, College Station, TX). Results meeting a 2-tailed *P* < 0.05 were considered statistically significant, and *P* < 0.1 was used to indicate a trend towards significance.

## Results

### Long-term prognosis of cancer patients according to PCI

In the primary analysis, the PCI + (n = 564; mean age 72 years) and PCI−/IHD+ (n = 3058; mean age 74 years) groups were compared. Baseline characteristics of the 2 groups are described in Table 1. The PCI + group had a lower prevalence of metastatic cancer, but a higher prevalence of ACS than the PCI-/IHD + group (33% vs. 15%). In terms of medication, PCI + group patients were more likely to receive β-blockers, statins, and ACE inhibitors. Furthermore, coronary risk factors such as smoking, hypertension, dyslipidaemia, and diabetes mellitus were more prevalent in the PCI + group.

**Fig. 2:**
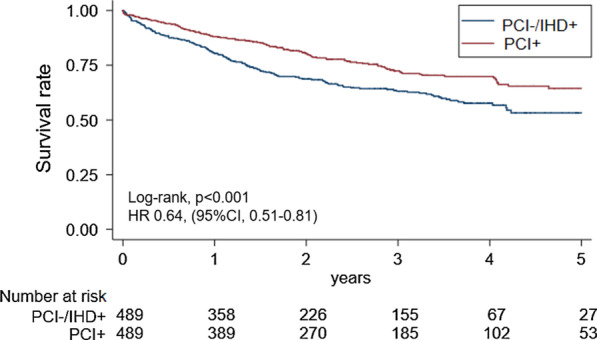
Kaplan–Meier analysis of all-cause mortality in the PCI + and PCI−/IHD + groups. After propensity score matching, Kaplan–Meier analysis was performed. The starting point of the survival analysis was the admission date for PCI or IHD. The PCI + group had better long-term prognosis compared to the PCI−/IHD + group. *PCI* percutaneous coronary intervention, *IHD* ischaemic heart disease, *HR* hazard ratio, *CI* confidence interval

To assess the effects of PCI, we compared the PCI + and PCI-/IHD + groups after propensity score matching. Adjusted variables were well-balanced after matching (standardised difference < 0.1). The PCI + group (n = 489) had significantly better prognoses than the PCI-/IHD + group (n = 489) (log-rank test, *P* < 0.001; Fig. 2). The Cox regression analysis with IPTW also found better prognoses in the PCI + group (n = 564) than in the PCI-/IHD + group (n = 3058) (hazard ratio [HR] 0.75, 95% confidence interval [CI] 0.59–0.96, *P* = 0.002]. Multivariable analysis showed that PCI was a significant independent predictor of all-cause mortality (HR 0.59, 95% CI 0.46–0.74, *P* < 0.001; see Additional file 1: Table S3). We also compared those who had undergone PCI or were diagnosed with IHD within 1.5 years (see Additional file 1: Table S4). The results also showed a better prognosis in the PCI + group (log-rank test, *P* = 0.011; see Additional file 1: Figure S2). Cox regression analysis with IPTW revealed better prognoses in the PCI + group (n = 394) than in the PCI−/IHD + group (n = 2621) (HR 0.75, 95% CI 0.57–0.97, *P* = 0.030).

**Fig. 3:**
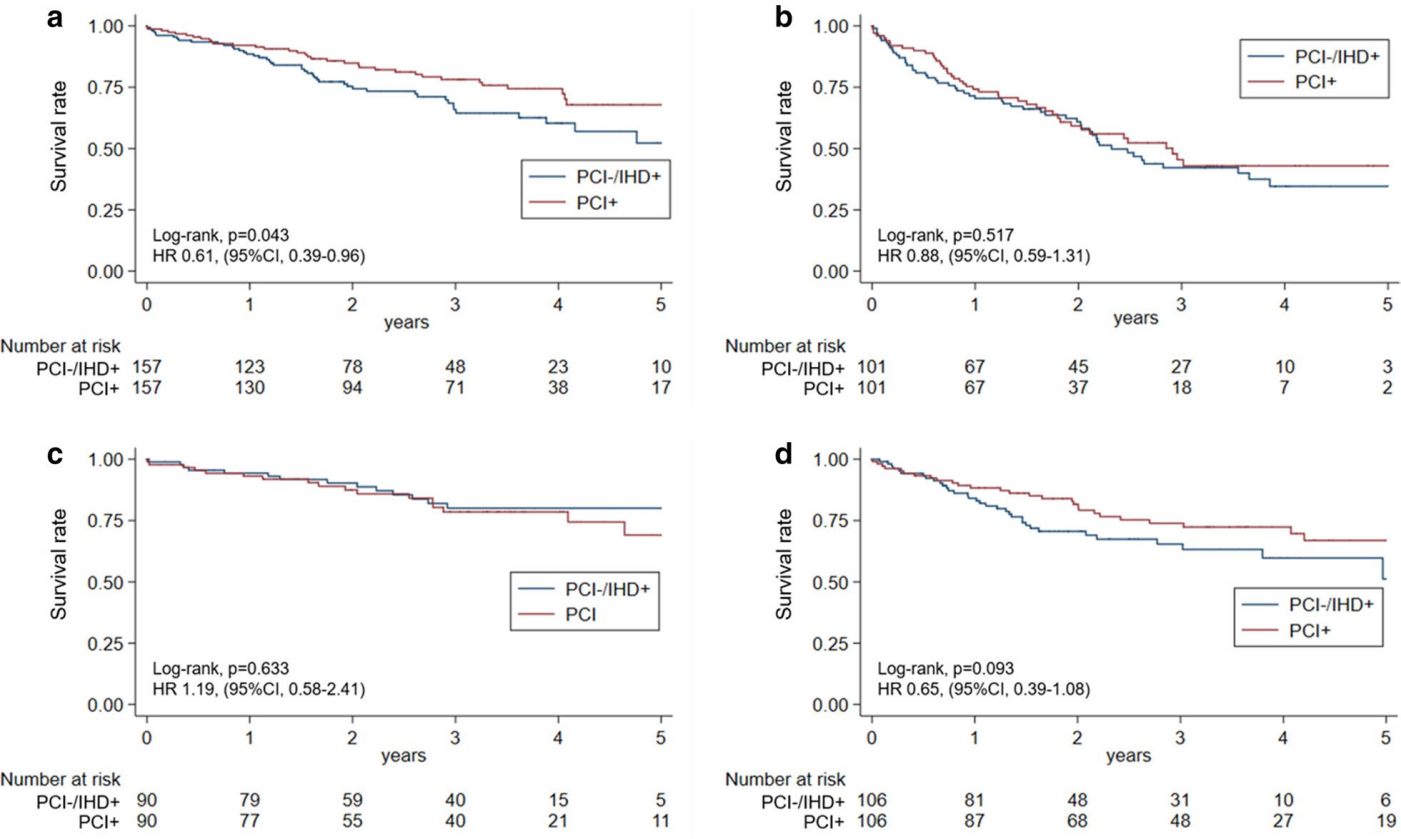
Kaplan–Meier analysis of all-cause mortality according to cancer type. Kaplan–Meier analysis of all-cause mortality in **a** colorectal, **b** lung, **c** prostate, and **d** gastric cancer patients was performed. Small cell carcinoma was considered a factor during propensity score matching of lung cancer patients. The starting point of the survival analysis was the admission date for PCI or IHD. *PCI* percutaneous coronary intervention, *IHD* ischaemic heart disease, *HR* hazard ratio, *CI* confidence interval

The effects of PCI on the long-term prognosis of each cancer type were also assessed (see Additional file 1: Tables S5 to S8). After propensity score matching, PCI + group patients with colorectal cancer had a significantly better prognosis (log-rank test, *P* = 0.043), while those with gastric cancer showed a trend toward improvement (log-rank test, *P* = 0.093) despite the relatively small number of patients (n = 157 and n = 106, respectively) (Fig. 3). Some variables had standardised difference > 0.1 in this propensity score-matched sample.

### Long-term prognosis of patients with IHD undergoing PCI and those without IHD

**Fig. 4:**
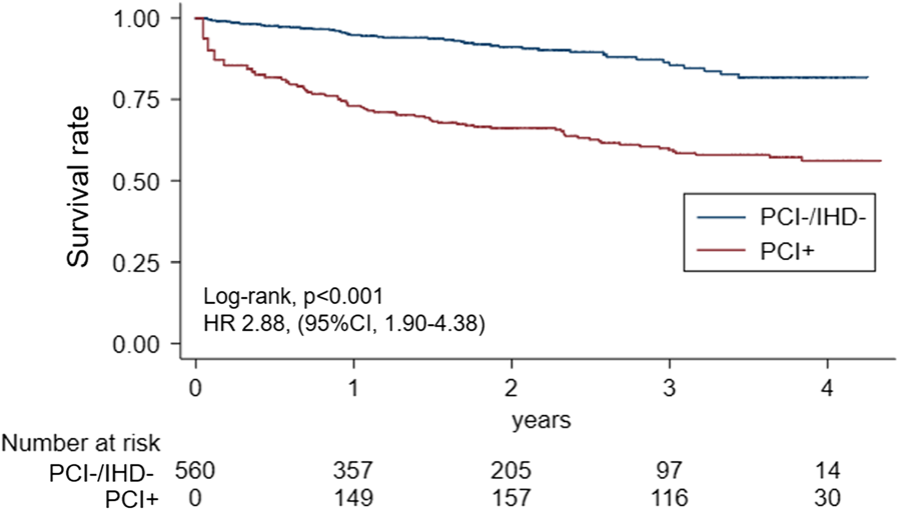
Kaplan–Meier analysis of all-cause mortality in the PCI + and PCI−/IHD− groups. The PCI + and PCI−/IHD− groups were propensity score-matched as was done in the prior analyses. Immortal time bias was adjusted for by considering PCI + patients as part of the PCI−/IHD− group during the period from cancer diagnosis to PCI admission, as the PCI + group had no patients at risk. The starting point of the survival analysis was the date of cancer diagnosis for both groups, but the PCI + group was allowed to contribute to the risk of the PCI−/IHD− group in the period before PCI. *PCI* percutaneous coronary intervention, *IHD* ischaemic heart disease, *HR* hazard ratio, *CI* confidence interval

Differences in all-cause mortality between patients who had undergone PCI (PCI + group, n = 282) and those who had had no documented IHD (PCI−/IHD− group, n = 27,392) (Table 2) were assessed. All-cause mortality between the PCI + (n = 0 at cancer diagnosis) and PCI−/IHD− groups (n = 560 at cancer diagnosis) were compared after adjusting for immortal time bias. Kaplan–Meier analysis of the propensity score-matched groups showed significantly higher all-cause mortality in the PCI+ group (log-rank test, *P* < 0.001) (Fig. 4). Multivariable analysis showed that PCI was an independent predictor of mortality (see Additional file 1: Table S9). Even after excluding ACS patients (see Additional file 1: Table S10), the PCI + group still showed higher mortality rates (log-rank test, *P* = 0.042) (see Additional file 1: Figure S4).

**Table 2:** Baseline characteristics of the PCI+ and PCI−/IHD− groups

	All patients (n = 27,676)		Entire cohort					Propensity score-matched sample				
			PCI+ (n = 282)		PCI−/IHD+ (n = 27,392)		SD^*^	PCI+ (n = 280)		PCI−/IHD+ (n = 280)		SD^*^
Age, mean ± standard deviance	70	± 10.2	73	± 6.9	70	± 10.2	0.822	73	± 7.0	73	± 8.4	0.027
Sex							0.444					0.077
Female	8563	(31)	37	(13)	8526	(31)		37	(13)	30	(11)	
Male	19,111	(69)	245	(87)	18,866	(69)		243	(87)	250	(89)	
Cancer type												
Colorectal cancer	9807	(35)	95	(34)	9712	(35)	0.037	95	(34)	97	(35)	0.015
Lung cancer	5997	(22)	68	(24)	5929	(22)	0.059	67	(24)	62	(22)	0.042
Prostate cancer	4317	(16)	55	(20)	4262	(16)	0.104	55	(20)	57	(20)	0.018
Gastric cancer	7553	(27)	64	(22)	7489	(27)	0.107	63	(22)	64	(23)	0.009
Cancer stage												
In situ	2569	(9)	21	(7)	2548	(9)	0.067	21	(7)	13	(5)	0.120
Localised	12,738	(46)	148	(52)	12,590	(46)	0.131	147	(53)	163	(58)	0.115
Regional to lymph nodes involved	2924	(11)	39	(14)	2885	(11)	0.101	39	(14)	31	(11)	0.086
Regional by direct extension	2818	(10)	36	(13)	2782	(10)	0.082	35	(12)	38	(14)	0.032
Distant site(s)/node(s) involved	5852	(21)	31	(11)	5821	(21)	0.282	31	(11)	32	(11)	0.011
Unknown	773	(3)	7	(3)	766	(3)	0.020	7	(3)	3	(1)	0.108
Barthel index score												
60–100	25,701	(93)	267	(95)	25,434	(93)	0.076	265	(95)	264	(94)	0.016
40–59	694	(2)	3	(1)	691	(2)	0.110	3	(1)	2	(1)	0.038
0–39	1279	(5)	12	(4)	1267	(5)	0.018	12	(4)	14	(5)	0.034
Overweight	6116	(22)	85	(30)	6031	(22)	0.186	84	(30)	85	(30)	0.008
Current or past smoking	14,138	(51)	168	(60)	13,970	(51)	0.173	166	(59)	165	(59)	0.007
Dyslipidemia	562	(2)	16	(6)	546	(2)	0.193	15	(5)	13	(5)	0.033
Hypertension	1393	(5)	29	(10)	1364	(5)	0.201	27	(10)	19	(7)	0.104
Diabetes mellitus	1137	(4)	34	(12)	1103	(4)	0.299	32	(11)	23	(8)	0.108
Chronic kidney disease	220	(1)	13	(5)	207	(1)	0.240	11	(4)	8	(3)	0.059
Congestive heart failure	250	(1)	11	(4)	239	(1)	0.199	9	(3)	4	(1)	0.119
Atrial fibrillation	308	(1)	0	(0)	308	(1)	0.151	0	(0)	0	(0)	NA
β-blocker	562	(2)	31	(11)	532	(2)	0.374	28	(10)	23	(8)	0.062
Statin	1251	(5)	46	(16)	1205	(4)	0.399	44	(16)	44	(16)	0.000
ACE inhibitor	301	(1)	9	(3)	292	(1)	0.065	9	(3)	2	(1)	0.181
ARB	1755	(6)	49	(17)	1707	(6)	0.343	46	(16)	43	(15)	0.029
Oral anti-coagulants	247	(1)	8	(3)	239	(1)	0.060	8	(3)	3	(1)	0.129
Acute coronary syndrome	–	–	81	(29)	–	–	NA	81	(29)	–	–	NA
Days from cancer diagnosis to PCI/IHD admission, median (IQR)	–	–	243	(92–543)	–	–	NA	242	(90–547)	–	–	NA
Chemo/radiation/hormonal therapy	10,220	(37)	97	(34)	10,123	(37)	0.079	97	(35)	94	(34)	0.023
Surgery or endoscopic resection	16,624	(60)	189	(67)	16,435	(60)	0.216	188	(67)	194	(69)	0.046

Data are presented as n (%) unless otherwise indicated

*ACE* angiotensin-converting enzyme, *ARB* angiotensin II receptor blocker, *IHD* ischaemic heart disease, *IQR* interquartile range, *NA* not available, *PCI* percutaneous coronary intervention, *SD* standardised difference, *NA* not available, *PCI+* cancer patients undergoing PCI, *PCI-/IHD* cancer patients without IHD and not undergoing PCI

## Discussion

### Impact of PCI on the survival of cancer patients with IHD

Our primary analysis suggests that PCI may lead to a better long-term prognosis in patients with certain cancers and IHD. Our results were verified using multiple tests such as IPTW and multivariable Cox proportional analysis. Additionally, similar results were observed after reducing the time interval from cancer diagnosis to index PCI or IHD from 3 to 1.5 years.

Cancer patients reportedly have a higher risk of cardiovascular events after PCI than non-cancer patients. Landes et al. [8] reported that cancer patients had higher rates of a composite of death, myocardial infarction, target lesion revascularisation (TLR), and CABG. Nakatsuma et al. [9] reported that the 5-year incidence of cardiac death was higher in cancer patients and that rates of definite or probable stent thrombosis also tended to be higher. A meta-analysis found that 1-year cardiovascular mortality after PCI was higher in cancer patients [11]. Taken together, these findings suggest that cancer can lead to the progression of atherosclerosis and increased cardiovascular mortality. In fact, Tabata et al. [13] reported that not only do cancer patients have higher 1-year TLR rates, but those with elevated high-sensitivity C-reactive protein levels also have higher overall cardiovascular event rates (cardiovascular death, non-fatal MI, unstable angina pectoris, TLR, non-TLR, and hospitalisation for heart failure decompensation). They speculated that increased inflammation in cancer patients might lead to the progression of coronary artery atherosclerosis. This may mean that cancer patients with IHD have a very high risk of cardiovascular events, which could explain why PCI and regular cardiology follow-up of our cancer patients reduced all-cause mortality.

We assessed the impact of PCI on each cancer type. However, despite the propensity score matching, the results were underpowered. Colorectal and gastric cancer patients in the PCI + group had significantly lower mortality and trends toward lower mortality, respectively, compared to PCI−/IHD + patients. This was consistent with the overall analysis. In contrast, no difference in mortality was observed between lung and prostate cancer patients in both groups. Since metastasis is more common in lung cancer patients, the advantage of PCI may be nullified by increased cancer lethality. In prostate cancer patients, a higher prevalence of a Barthel Index of 40–59, treatment with oral anticoagulants, and chemo/radiation/hormonal therapy, which were not sufficiently balanced after propensity score matching, might have affected the results. Furthermore, since prostate cancer has low lethality, there may be fewer reasons to forego PCI. Therefore, one possible explanation is that PCI-/IHD- group patients with prostate cancer might have had relatively low-risk IHD that did not require PCI. One of the major concerns with PCI is post-procedural bleeding. It has been shown that gastrointestinal cancer patients have higher rates of gastrointestinal bleeding after PCI [24, 25]. Our results suggest that the advantages of PCI might outweigh bleeding risk.

The present study had several confounders and limitations due to the nature of its retrospective cancer-based cohort. Indeed, our study lacked an analysis of some significant cardiovascular-related factors as unmeasured confounders. For example, we could not determine the reason why PCI−/IHD + group patients did not undergo PCI. Nevertheless, the cancer-related background characteristics were very detailed in our study. Although it may be difficult to directly apply our study’s findings to daily practice, there are still some clinical implications. Cardiologists may hesitate to offer aggressive cardiovascular interventions to cancer patients considering their prognosis. However, according to our study, there should be less prevarication in performing the necessary intervention for IHD under the proper indication. In addition, cardiovascular intervention and outpatient follow-up by cardiologists might improve the prognosis of cancer patients with IHD. Furthermore, our study will encourage and provide a basis for further clinical trials or prospective investigations for better understanding of this issue in cardio-oncology.

### Impact of IHD and PCI on the survival of cancer patients

Secondary analysis showed that cancer patients undergoing PCI had higher mortality compared to those who had no history of IHD. As shown in Fig. 4, the difference between the two groups increased over the first few months. Roule et al. reported that cancer patients undergoing PCI for ACS have higher rates of all-cause (relative risk [RR] 2.62, 95% CI 1.2–5.73) and cardiac deaths (RR 2.44, 95% CI 1.73–3.4,) compared to non-cancer patients [12]. Although their study population differed from ours, the results of the two investigations are consistent. In order to exclude the potential impact of ACS prevalence on the short-term prognosis, we analysed the mortality only in patients undergoing PCI for stable IHD as a sensitivity analysis. Similar to other results, long-term mortality was worse in the subgroup of patients who underwent PCI for stable IHD. This result may also be related to the elevated inflammatory state mentioned earlier [8, 9, 11, 13]. The PCI + group patients could have had a higher risk of cardiovascular events, including cardiac death, compared to the PCI−/IHD− group patients.

### Limitations

Our study had several limitations. First, since this was a retrospective registry-based cohort study, we could not adjust for all confounders. Second, we could not identify a history of coronary artery diseases or any related treatment that occurred before the beginning of administrative data collection in 2010. Third, cause of death data (e.g., cardiovascular or cancer-related) and PCI procedural variables (i.e., type of stent used) were not available. In addition, we did not have ischaemic parameters and disease extent data for IHD patients. Fourth, despite the use of a large cancer registry, the number of patients we identified who had undergone PCI was relatively small. Fifth, a substantial number of cancer patients were not hospitalised after being definitively diagnosed with cancer; therefore, our secondary analysis lacked ADL data (Barthel Index score). Sixth, the use of antiplatelet therapy was not assessed. Because antiplatelet treatment was contraindicated for most of the patients in the PCI−/IHD + group, antiplatelet therapy rates were not appropriate covariates for propensity score matching. Thus, it should be counted as a factor “not prevalent in PCI patients” in this study. To address these limitations, more studies are needed.

## Conclusion

PCI might improve the long-term prognosis of cancer patients with IHD. However, cancer patients who undergo PCI could have significantly worse long-term prognoses than those without IHD. Since the present study has some unmeasured confounders and limitations, further prospective investigations of this important cardio-oncology topic are needed.

## Supplementary Information

**Additional file 1.** Supplementary tables and figures.

## Data Availability

The datasets used and analysed during the current study are available from the corresponding author upon reasonable request.

## Abbreviations

ACE: Angiotensin-converting enzyme
ACS: Acute coronary syndrome
ADL: Activities of daily living
ARB: Angiotensin II receptor blocker
CABG: Coronary artery bypass grafting
CAD: Coronary artery diseases
CI: Confidence interval
DPC: Diagnosis Procedure Combination Per-diem Payment System
HR: Hazard ratio
ICD-10: International Classification of Diseases, Tenth Revision
ICD-O-3: International Classification of Diseases for Oncology, Third Edition
IHD: Ischaemic heart disease
IPTW: Inverse probability of treatment weighting
IQR: Interquartile range
NA: Not available
OCR: Osaka Cancer Registry
OMI: Old myocardial infarction
PCI: Percutaneous coronary intervention
RR: Relative risk
TLR: Target lesion revascularisation
