# Supplementary material for: Multicentre cohort study of the impact of percutaneous coronary intervention on patients with concurrent cancer and ischaemic heart disease
Source: BMC Cardiovasc Disord. 2021 Apr 13;21:177. doi: 10.1186/s12872-021-01968-w (PMC8045293; doi:10.1186/s12872-021-01968-w)
Supplement: Supplementary file 1 — Additional file 1. Supplementary tables and figures. [file 12872_2021_1968_MOESM1_ESM.docx]

**Additional File1: Supplemental Data**

Manuscript Title: Multicentre cohort study of the impact of percutaneous coronary intervention on patients with concurrent cancer and ischaemic heart disease

Authors: Tatsuya Nishikawa*^1^, MD, PhD; Toshitaka Morishima^2^, MD, PhD; Sumiyo Okawa^2^, PhD; Yuki Fujii^1^, MD; Tomoyuki Otsuka^3^, MD, PhD; Toshihiro Kudo, MD, PhD; Takeshi Fujita, MD; Risa Kamada^1^, MD; Taku Yasui^1^, MD, PhD; Wataru Shioyama^1^, MD, PhD; Toru Oka^1^, MD, PhD; Takahiro Tabuchi^2^, MD, PhD; Masashi Fujita*^1^, MD, PhD; Isao Miyashiro^2^, MD, PhD

^1^ Department of Onco-Cardiology, Osaka International Cancer Institute, Osaka, Japan

^2^ Cancer Control Centre, Osaka International Cancer Institute, Osaka, Japan

^3^ Department of Clinical Oncology, Osaka International Cancer Institute, Osaka, Japan

Corresponding Authors: Masashi Fujita and Tatsuya Nishikawa


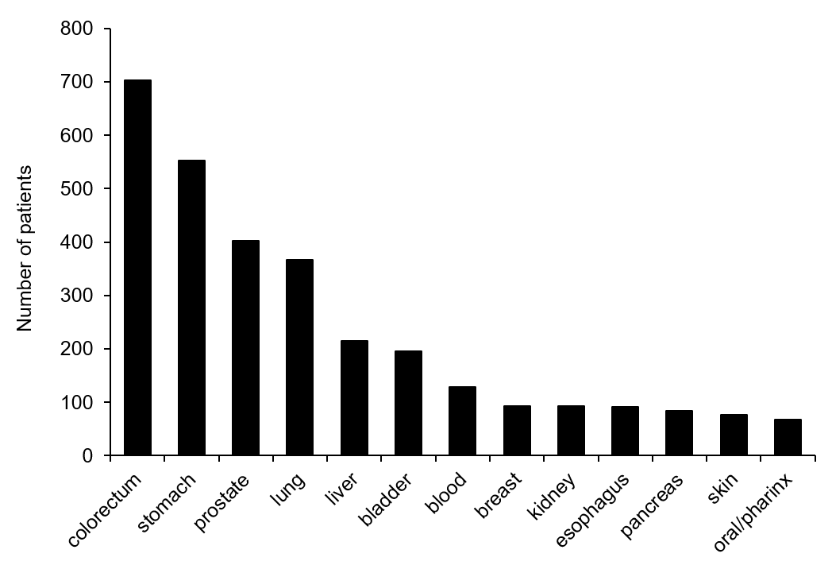


**Supplemental Figure S1** **Total number of patients with PCI in the Osaka Cancer Registry**

The total number of patients who had PCI for each cancer type in the Osaka Cancer Registry is shown.

PCI, percutaneous coronary intervention

**Supplemental Table S1** **Definitions of diagnoses and corresponding ICD-10 codes**

| Variates | ICD-10 |
| --- | --- |
| Ischaemic heart disease | I20.x, I21.x, I25.5 |
| Dyslipidaemia | E78.0, E78.1, E78.2, E78.3, E78.4, E78.5 |
| Hypertension | I10.x, I11.x, I12.x, I13.x |
| Diabetes mellitus | E10.x, E11.x, E12.x, E13.x, E14.x |
| Chronic kidney disease | N18.x |
| Congestive heart failure | I15.0 |
| Acute coronary syndrome | I20.0, I21.x |
| Old myocardial infarction | I25.2 |
| Atrial fibrillation | I48.x |

ICD-10, International Classification of Diseases, 10th Revision

**Supplemental Table S2 Medications considered in the analysis**

| Medications | Generic name |
| --- | --- |
| Statins | Atorvastatin, Rosuvastatin, Pitavastatin, Fluvastatin, Pravastatin |
| β-blocker | Carvedilol, Bisoprolol |
| Angiotensin-converting enzyme inhibitor | Captopril, Enalapril, Alacepril, Delapril, Cilazapril, Benazepril, ‎Imidapril, Temocapril, Quinapril, Trandolapril, Perindopril |
| Angiotensin II receptor blocker | ‎Losartan, Candesartan, Valsartan, Telmisartan, Olmesartan, ‎Irbesartan, Azilsartan |
| Oral anti-coagulants | Warfarin, Dabigatran, Apixaban, Rivaroxaban, Edoxaban |

**Supplemental** **Table S3** **Multivariable analysis of mortality with propensity score matching for the primary analysis**

|  | **HR** | **[95% CI]** | **P-value** |
| --- | --- | --- | --- |
| PCI | 0.59 | [0.46–0.74] | <0.001 |
| Age | 1.06 | [1.04–1.08] | <0.001 |
| Sex |  |  |  |
| Male | Ref |  |  |
| Female | 0.92 | [0.67–1.27] | 0.619 |
| Cancer type |  |  |  |
| Colorectal cancer | Ref |  |  |
| Lung cancer | 2.38 | [1.75–3.23] | <0.001 |
| Prostate cancer | 0.63 | [0.41–0.96] | 0.033 |
| Gastric cancer | 1.35 | [0.95–1.92] | 0.096 |
| Cancer stage |  |  |  |
| In situ | Ref |  |  |
| Localised | 0.91 | [0.47–1.77] | 0.777 |
| Regional to lymph node involvement | 2.37 | [1.20–4.65] | 0.012 |
| Regional by direct extension | 2.25 | [1.13–4.48] | 0.020 |
| Distant site(s)/node(s) involved | 6.95 | [3.48–13.91] | <0.001 |
| Unknown | 5.37 | [2.45–11.75] | <0.001 |
| Barthel Index |  |  |  |
| 60–100 | Ref |  |  |
| 40–59 | 1.66 | [0.96–2.88] | 0.069 |
| 0–39 | 1.95 | [1.38–2.74] | <0.001 |
| Acute coronary syndrome | 1.08 | [0.83–1.40] | 0.548 |
| Interval from cancer diagnosis to PCI/IHD admission, days^*^ | 1.00 | [0.9997–1.0005] | 0.740 |

^*^Expressed as the hazard ratio per one-day increase.

CI, confidence interval; HR, hazard ratio; IHD, ischaemic heart disease; PCI, percutaneous coronary intervention; Ref, reference


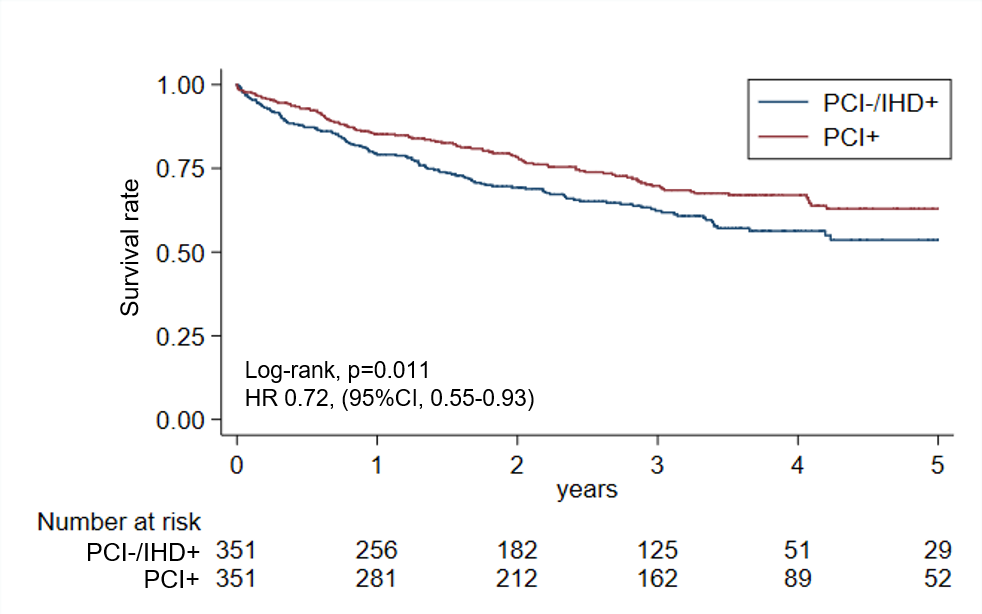


**Supplemental Figure S2 Kaplan-Meier analysis of all-cause mortality for the PCI+ group and the PCI-/IHD+ group**

The PCI+ group contained only those who had PCI within 1.5 years of cancer diagnosis. Moreover, the PCI-/IHD+ group contained only those who had a diagnosis of IHD within 1.5 years of cancer prognosis.

PCI, percutaneous coronary intervention; IHD, ischaemic heart disease; HR, hazard ratio; CI, confidence interval

**Supplemental Table S4** **Baseline characteristics for sensitivity analysis of the primary analysis, as shown in Supplemental Figure 1**

|  |  | |  | |  | | Entire cohort | | | | | | | | | Propensity score-matched sample | | | | | | |  |  |
| --- | --- | --- | --- | --- | --- | --- | --- | --- | --- | --- | --- | --- | --- | --- | --- | --- | --- | --- | --- | --- | --- | --- | --- | --- |
|  | | All patients (n=3015) | | | | | | PCI+ (n=394) | | | | PCI-/IHD+ (n=2621) | | | SD^*^ | PCI+ (n=352) | | | PCI-/IHD+ (n=352) | | | SD^*^ |  |  |
| Age, mean ± standard deviance | | 74 | | ± | | 7.9 | | | 73 | ± | 7.1 | 74 | ± | 8.0 | 0.204 | 73 | ± | 7.1 | 73 | ± | 7.8 | 0.011 |  |  |
| Sex | |  | |  | |  | | |  |  |  |  |  |  | 0.234 |  |  |  |  |  |  | 0.024 |  |  |
| Female | | 470 | | (22) | | | | 57 | | (14) | | 613 | (23) | |  | 52 | (15) | | 49 | (14) | |  |  |  |
| Male | | 2345 | | (78) | | | | 337 | | (86) | | 2008 | (77) | |  | 300 | (85) | | 303 | (86) | |  |  |  |
| Cancer type | |  | |  | | | |  | |  | |  |  | |  |  |  | |  |  | |  |  |  |
| Colorectal cancer | | 958 | | (32) | | | | 134 | | (34) | | 824 | (32) | | 0.055 | 118 | (34) | | 121 | (34) | | 0.018 |  |  |
| Lung cancer | | 803 | | (27) | | | | 90 | | (23) | | 713 | (27) | | 0.101 | 84 | (24) | | 91 | (26) | | 0.046 |  |  |
| Prostate cancer | | 370 | | (12) | | | | 82 | | (21) | | 288 | (11) | | 0.271 | 68 | (19) | | 59 | (17) | | 0.067 |  |  |
| Gastric cancer | | 884 | | (29) | | | | 88 | | (22) | | 796 | (30) | | 0.183 | 82 | (23) | | 81 | (23) | | 0.007 |  |  |
| Cancer stage | |  | |  | | | |  | |  | |  |  | |  |  |  | |  |  | |  |  |  |
| In situ | | 181 | | (6) | | | | 30 | | (8) | | 151 | (6) | | 0.074 | 26 | (7) | | 27 | (8) | | 0.011 |  |  |
| Localized | | 1498 | | (50) | | | | 189 | | (48) | | 1309 | (50) | | 0.039 | 171 | (49) | | 171 | (49) | | 0.000 |  |  |
| Regional to lymph nodes involved | | 410 | | (14) | | | | 53 | | (13) | | 357 | (14) | | 0.005 | 46 | (13) | | 53 | (15) | | 0.057 |  |  |
| Regional by direct extension | | 330 | | (11) | | | | 58 | | (15) | | 272 | (10) | | 0.131 | 53 | (15) | | 47 | (13) | | 0.049 |  |  |
| Distant site(s)/node(s) involved | | 521 | | (17) | | | | 47 | | (12) | | 474 | (18) | | 0.173 | 45 | (13) | | 41 | (12) | | 0.035 |  |  |
| Unknown | | 75 | | (2) | | | | 17 | | (4) | | 58 | (2) | | 0.118 | 11 | (3) | | 13 | (3) | | 0.031 |  |  |
| Barthel Index score | |  | |  | | | |  | |  | |  |  | |  |  |  | |  |  | |  | |  |
| 60-100 | | 2702 | | (90) | | | | 345 | | (88) | | 2357 | (90) | | 0.075 | 308 | (88) | | 308 | (88) | | 0.000 |  |  |
| 40-59 | | 116 | | (4) | | | | 13 | | (3) | | 103 | (4) | | 0.034 | 12 | (4) | | 13 | (4) | | 0.015 |  |  |
| 0-39 | | 197 | | (6) | | | | 36 | | (9) | | 161 | (6) | | 0.113 | 32 | (9) | | 31 | (9) | | 0.010 |  |  |
| Overweight | | 781 | | (26) | | | | 108 | | (27) | | 673 | (26) | | 0.048 | 98 | (28) | | 83 | (24) | | 0.097 |  |  |
| Current or past smoking | | 1667 | | (55) | | | | 235 | | (60) | | 1432 | (55) | | 0.119 | 212 | (60) | | 211 | (60) | | 0.006 |  |  |
| Dyslipidemia | | 791 | | (26) | | | | 205 | | (52) | | 586 | (22) | | 0.660 | 172 | (49) | | 163 | (43) | | 0.051 |  |  |
| Hypertension | | 1472 | | (49) | | | | 258 | | (65) | | 1214 | (46) | | 0.393 | 332 | (63) | | 225 | (64) | | 0.024 |  |  |
| Diabetes mellitus | | 940 | | (31) | | | | 178 | | (45) | | 762 | (29) | | 0.317 | 152 | (43) | | 152 | (43) | | 0.000 |  |  |
| Chronic kidney disease | | 187 | | (6) | | | | 31 | | (8) | | 156 | (6) | | 0.082 | 27 | (8) | | 25 | (7) | | 0.022 |  |  |
| Congestive heart failure | | 472 | | (16) | | | | 105 | | (27) | | 367 | (14) | | 0.308 | 85 | (24) | | 85 | (24) | | 0.000 |  |  |
| Atrial fibrillation | | 229 | | (8) | | | | 39 | | (10) | | 190 | (7) | | 0.085 | 33 | (9) | | 37 | (11) | | 0.038 |  |  |
| β−blocker | | 659 | | (22) | | | | 149 | | (38) | | 510 | (19) | | 0.433 | 124 | (35) | | 117 | (33) | | 0.042 |  |  |
| Statin | | 868 | | (29) | | | | 199 | | (51) | | 669 | (26) | | 0.557 | 169 | (48) | | 174 | (49) | | 0.028 |  |  |
| ACE inhibitor | | 238 | | (8) | | | | 66 | | (17) | | 172 | (7) | | 0.331 | 47 | (13) | | 50 | (14) | | 0.025 |  |  |
| ARB | | 926 | | (31) | | | | 134 | | (34) | | 732 | (30) | | 0.098 | 118 | (34) | | 124 | (35) | | 0.036 |  |  |
| Oral anti-coagulants | | 348 | | (12) | | | | 40 | | (10) | | 308 | (12) | | 0.040 | 36 | (10) | | 41 | (12) | | 0.045 |  |  |
| Acute coronary syndrome | | 526 | | (17) | | | | 135 | | (34) | | 391 | (15) | | 0.455 | 105 | (30) | | 114 | (32) | | 0.055 |  |  |
| Days from cancer diagnosis to PCI/IHD admission, median (IQR) | | 58 | | (25-173) | | | | 156 | | (57-317) | | 54 | (23-133) | | 0.557 | 150 | (53-283) | | 98 | (51-354) | | 0.019 |  |  |
| Chemo/radiation/hormonal therapy | | 1043 | | (35) | | | | 124 | | (31) | | 919 | (35) | | 0.077 | 111 | (32) | | 112 | (32) | | 0.006 |  |  |
| Surgery or endoscopic resection | | 1893 | | (63) | | | | 243 | | (32) | | 1650 | (63) | | 0.019 | 221 | (63) | | 221 | (63) | | 0.000 |  |  |

Data are presented as n (%) unless otherwise indicated.

ACE, angiotensin-converting enzyme; ARB, angiotensin II receptor blocker; IHD, ischaemic heart disease; IQR, interquartile range; PCI, percutaneous coronary intervention; SD, standardized difference

**Supplemental Table S5** **Baseline characteristics for colorectal cancer as a subgroup analysis in the primary analysis**

|  |  | |  | |  | | Entire cohort | | | | | | | Propensity score-matched sample | | | | | | |
| --- | --- | --- | --- | --- | --- | --- | --- | --- | --- | --- | --- | --- | --- | --- | --- | --- | --- | --- | --- | --- |
|  | | All patients (n=1165) | | | | | PCI+ (n=195) | | | PCI-/IHD+ (n=970) | | | SD^*^ | PCI+ (n=157) | | | PCI-/IHD+ (n=157) | | | SD^*^ |
| Age, mean ± standard deviance | | 74 | | ± | | 8.3 | 72 | ± | 7.9 | 74 | ± | 8.4 | 0.241 | 73 | ± | 7.6 | 73 | ± | 8.8 | 0.012 |
| Sex | |  | |  | |  |  |  |  |  |  |  | 0.281 |  |  |  |  |  |  | 0.060 |
| Female | | 361 | | (31) | | | 40 | (21) | | 321 | (33) | |  | 122 | (78) | | 39 | (25) | |  |
| Male | | 804 | | (69) | | | 155 | (79) | | 649 | (67) | |  | 35 | (22) | | 118 | (75) | |  |
| Cancer stage | |  | |  | | |  |  | |  |  | |  |  |  | |  |  | |  |
| In situ | | 251 | | (22) | | | 48 | (25) | | 203 | (21) | | 0.088 | 39 | (25) | | 42 | (27) | | 0.044 |
| Localized | | 481 | | (41) | | | 72 | (37) | | 409 | (42) | | 0.107 | 60 | (38) | | 59 | (38) | | 0.013 |
| Regional to lymph nodes involved | | 188 | | (16) | | | 36 | (18) | | 152 | (16) | | 0.074 | 28 | (18) | | 25 | (16) | | 0.051 |
| Regional by direct extension | | 96 | | (8) | | | 16 | (8) | | 80 | (8) | | 0.002 | 13 | (8) | | 18 | (11) | | 0.107 |
| Distant site(s)/node(s) involved | | 130 | | (11) | | | 16 | (8) | | 114 | (12) | | 0.119 | 13 | (8) | | 8 | (5) | | 0.128 |
| Unknown | | 19 | | (2) | | | 7 | (4) | | 12 | (1) | | 0.154 | 4 | (3) | | 5 | (3) | | 0.038 |
| Barthel Index score | |  | |  | | |  |  | |  |  | |  |  |  | |  |  | |  |
| 60-100 | | 1001 | | (86) | | | 162 | (83) | | 839 | (86) | | 0.095 | 134 | (85) | | 132 | (84) | | 0.035 |
| 40-59 | | 61 | | (5) | | | 10 | (5) | | 51 | (5) | | 0.006 | 8 | (5) | | 9 | (6) | | 0.028 |
| 0-39 | | 103 | | (9) | | | 23 | (12) | | 80 | (9) | | 0.118 | 15 | (10) | | 16 | (10) | | 0.021 |
| Overweight | | 339 | | (29) | | | 58 | (30) | | 281 | (29) | | 0.024 | 46 | (29) | | 46 | (29) | | 0.000 |
| Current or past smoking | | 491 | | (42) | | | 90 | (46) | | 401 | (41) | | 0.096 | 71 | (45) | | 63 | (40) | | 0.103 |
| Dyslipidemia | | 335 | | (29) | | | 103 | (53) | | 232 | (24) | | 0.633 | 73 | (47) | | 76 | (48) | | 0.038 |
| Hypertension | | 613 | | (53) | | | 135 | (69) | | 478 | (49) | | 0.419 | 98 | (62) | | 98 | (62) | | 0.040 |
| Diabetes mellitus | | 389 | | (33) | | | 91 | (47) | | 298 | (31) | | 0.320 | 69 | (44) | | 69 | (44) | | 0.013 |
| Chronic kidney disease | | 97 | | (8) | | | 22 | (11) | | 75 | (8) | | 0.125 | 16 | (10) | | 15 | (10) | | 0.021 |
| Congestive heart failure | | 227 | | (19) | | | 53 | (27) | | 174 | (18) | | 0.228 | 41 | (26) | | 44 | (28) | | 0.043 |
| Atrial fibrillation | | 133 | | (11) | | | 22 | (11) | | 111 | (11) | | 0.001 | 18 | (11) | | 19 | (12) | | 0.020 |
| β−blocker | | 291 | | (25) | | | 96 | (49) | | 195 | (20) | | 0.633 | 65 | (41) | | 68 | (43) | | 0.039 |
| Statin | | 388 | | (33) | | | 115 | (59) | | 273 | (28) | | 0.667 | 83 | (53) | | 80 | (51) | | 0.038 |
| ACE inhibitor | | 104 | | (9) | | | 42 | (22) | | 62 | (6) | | 0.452 | 26 | (17) | | 23 | (15) | | 0.053 |
| ARB | | 385 | | (33) | | | 86 | (44) | | 299 | (31) | | 0.286 | 67 | (43) | | 67 | (43) | | 0.000 |
| Oral anti-coagulants | | 184 | | (16) | | | 28 | (14) | | 156 | (16) | | 0.044 | 25 | (16) | | 29 | (19) | | 0.067 |
| Acute coronary syndrome | | 220 | | (19) | | | 68 | (35) | | 152 | (16) | | 0.449 | 42 | (27) | | 40 | (25) | | 0.029 |
| Days from cancer diagnosis to PCI/IHD admission, median (IQR) | | 77 | | (30-431) | | | 334 | (96-618) | | 61 | (27-366) | | 0.577 | 282 | (89-544) | | 316 | (43-646) | | 0.054 |
| Chemo/radiation/hormonal therapy | | 214 | | (18) | | | 37 | (19) | | 177 | (18) | | 0.024 | 30 | (19) | | 24 | (15) | | 0.101 |
| Surgery or endoscopic resection | | 985 | | (85) | | | 165 | (85) | | 820 | (85) | | 0.002 | 133 | (85) | | 133 | (85) | | 0.000 |

Data are presented as n (%) unless otherwise indicated.

ACE, angiotensin-converting enzyme; ARB, angiotensin II receptor blocker; IHD, ischaemic heart disease; IQR, interquartile range; PCI, percutaneous coronary intervention; SD, standardized difference

**Supplemental Table S6 Baseline characteristics for lung cancer as a subgroup analysis in the primary analysis**

|  |  | |  | |  | | Entire cohort | | | | | | | | | | Propensity score-matched sample | | | | | | | | |
| --- | --- | --- | --- | --- | --- | --- | --- | --- | --- | --- | --- | --- | --- | --- | --- | --- | --- | --- | --- | --- | --- | --- | --- | --- | --- |
|  | All patients (n=910) | | | | | | PCI+ (n=115) | | | | PCI-/IHD+ (n=795) | | | | SD^*^ | | PCI+ (n=101) | | | PCI-/IHD+ (n=101) | | | | SD^*^ | |
| Age, mean ± standard deviance | | 73 | | ± | | 7.7 | | 73 | ± | 6.5 | | 74 | ± | 7.8 | | 0.144 | 73 | ± | 6.2 | | 73 | ± | 8.1 | | 0.047 |
| Sex | |  | |  | |  | |  |  |  | |  |  |  | | 0.187 |  |  |  | |  |  |  | | 0.026 |
| Female | | 214 | | (24) | | | | 19 | (17) | | | 195 | (25) | | |  | 18 | (18) | | | 17 | (17) | | |  |
| Male | | 696 | | (76) | | | | 96 | (83) | | | 600 | (75) | | |  | 83 | (82) | | | 84 | (83) | | |  |
| Cancer stage | |  | |  | | | |  |  | | |  |  | | |  |  |  | | |  |  | | |  |
| In situ | | 1 | | (0) | | | | 0 | (0) | | | 1 | (0) | | | 0.050 | 0 | (0) | | | 0 | (0) | | | NA |
| Localized | | 364 | | (40) | | | | 57 | (50) | | | 307 | (39) | | | 0.222 | 50 | (50) | | | 53 | (52) | | | 0.059 |
| Regional to lymph nodes involved | | 148 | | (16) | | | | 20 | (17) | | | 128 | (16) | | | 0.035 | 16 | (16) | | | 17 | (17) | | | 0.027 |
| Regional by direct extension | | 99 | | (11) | | | | 15 | (13) | | | 84 | (11) | | | 0.077 | 13 | (13) | | | 9 | (9) | | | 0.127 |
| Distant site(s)/node(s) involved | | 270 | | (30) | | | | 21 | (18) | | | 249 | (31) | | | 0.306 | 21 | (20) | | | 22 | (22) | | | 0.024 |
| Unknown | | 28 | | (3) | | | | 2 | (2) | | | 26 | (3) | | | 0.098 | 1 | (1) | | | 0 | (0) | | | 0.141 |
| Small cell carcinoma | | 104 | | (11) | | | | 6 | (5) | | | 98 | (12) | | | 0.253 | 6 | (6) | | | 6 | (6) | | | 0.000 |
| Barthel Index score | |  | |  | | | |  |  | | |  |  | | |  |  |  | | |  |  | | |  |
| 60-100 | | 807 | | (89) | | | | 104 | (90) | | | 703 | (88) | | | 0.065 | 91 | (90) | | | 93 | (92) | | | 0.070 |
| 40-59 | | 41 | | (4) | | | | 4 | (4) | | | 37 | (5) | | | 0.060 | 3 | (3) | | | 2 | (2) | | | 0.064 |
| 0-39 | | 62 | | (7) | | | | 7 | (6) | | | 55 | (7) | | | 0.034 | 7 | (7) | | | 6 | (6) | | | 0.040 |
| Overweight | | 223 | | (25) | | | | 32 | (28) | | | 191 | (24) | | | 0.103 | 27 | (27) | | | 23 | (23) | | | 0.091 |
| Current or past smoking | | 672 | | (74) | | | | 89 | (77) | | | 583 | (73) | | | 0.101 | 79 | (78) | | | 79 | (78) | | | 0.000 |
| Dyslipidemia | | 275 | | (30) | | | | 63 | (55) | | | 212 | (27) | | | 0.628 | 52 | (51) | | | 47 | (47) | | | 0.099 |
| Hypertension | | 470 | | (52) | | | | 78 | (68) | | | 392 | (49) | | | 0.382 | 66 | (65) | | | 66 | (65) | | | 0.000 |
| Diabetes mellitus | | 299 | | (33) | | | | 50 | (43) | | | 249 | (31) | | | 0.222 | 42 | (42) | | | 40 | (40) | | | 0.040 |
| Chronic kidney disease | | 54 | | (6) | | | | 7 | (6) | | | 47 | (6) | | | 0.014 | 5 | (5) | | | 5 | (5) | | | 0.000 |
| Congestive heart failure | | 155 | | (17) | | | | 37 | (32) | | | 118 | (15) | | | 0.396 | 28 | (28) | | | 20 | (20) | | | 0.186 |
| Atrial fibrillation | | 72 | | (8) | | | | 17 | (15) | | | 55 | (7) | | | 0.240 | 15 | (15) | | | 16 | (16) | | | 0.027 |
| β−blocker | | 203 | | (23) | | | | 44 | (38) | | | 159 | (20) | | | 0.431 | 38 | (38) | | | 31 | (31) | | | 0.146 |
| Statin | | 281 | | (31) | | | | 61 | (53) | | | 220 | (28) | | | 0.564 | 51 | (51) | | | 51 | (51) | | | 0.000 |
| ACE inhibitor | | 64 | | (7) | | | | 22 | (19) | | | 42 | (5) | | | 0.444 | 16 | (16) | | | 14 | (14) | | | 0.055 |
| ARB | | 301 | | (33) | | | | 45 | (39) | | | 256 | (32) | | | 0.165 | 38 | (38) | | | 34 | (34) | | | 0.082 |
| Oral anti-coagulants | | 101 | | (11) | | | | 14 | (12) | | | 87 | (11) | | | 0.048 | 14 | (14) | | | 14 | (14) | | | 0.000 |
| Acute coronary syndrome | | 145 | | (16) | | | | 41 | (36) | | | 104 | (13) | | | 0.564 | 35 | (35) | | | 32 | (32) | | | 0.063 |
| Days from cancer diagnosis to PCI/IHD admission, median (IQR) | | 62 | | (24-247) | | | | 193 | (68-443) | | | 56 | (22-209) | | | 0.475 | 178 | (54-415) | | | 89 | (31-542) | | | 0.054 |
| Chemo/radiation/hormonal therapy | | 484 | | (53) | | | | 39 | (34) | | | 445 | (56) | | | 0.453 | 37 | (37) | | | 26 | (26) | | | 0.236 |
| Surgery or endoscopic resection | | 398 | | (44) | | | | 73 | (63) | | | 325 | (41) | | | 0.481 | 63 | (62) | | | 71 | (70) | | | 0.167 |

Data are presented as n (%) unless otherwise indicated.

ACE, angiotensin-converting enzyme; ARB, angiotensin II receptor blocker; IHD, ischaemic heart disease; IQR, interquartile range; NA, not available; PCI, percutaneous coronary intervention; SD, standardized difference

**Supplemental Table S7 Baseline characteristics for prostate cancer as a subgroup analysis in the primary analysis**

|  |  | |  |  | | | Entire cohort | | | | | | | | Propensity score-matched sample | | | | | | |
| --- | --- | --- | --- | --- | --- | --- | --- | --- | --- | --- | --- | --- | --- | --- | --- | --- | --- | --- | --- | --- | --- |
|  | | All patients (n=505) | | | | | | PCI+ (n=124) | | | PCI-/IHD+ (n=381) | | | SD^*^ | PCI+ (n=90) | | | PCI-/IHD+ (n=90) | | | SD^*^ |
| Age, mean ± standard deviance | | 73 | | | ± | 6.5 | | 73 | ± | 5.6 | 74 | ± | 6.8 | 0.185 | 73 | ± | 5.6 | 73 | ± | 6.8 | 0.029 |
| Sex | |  | | |  |  | |  |  |  |  |  |  | NA |  |  |  |  |  |  | NA |
| Female | | 0 | | | (0) | | | 0 | (0) | | 0 | (0) | |  | 0 | (0) | | 0 | (0) | |  |
| Male | | 505 | | | (100) | | | 124 | (100) | | 381 | (100) | |  | 90 | (100) | | 90 | (100) | |  |
| Cancer stage | |  | | |  | | |  |  | |  |  | |  |  |  | |  |  | |  |
| In situ | | 0 | | | (0) | | | 0 | (0) | | 0 | (0) | | NA | 0 | (0) | | 0 | (0) | | NA |
| Localized | | 307 | | | (61) | | | 70 | (56) | | 237 | (62) | | 0.117 | 51 | (57) | | 54 | (60) | | 0.068 |
| Regional to lymph nodes involved | | 6 | | | (1) | | | 1 | (1) | | 5 | (1) | | 0.049 | 1 | (1) | | 2 | (2) | | 0.087 |
| Regional by direct extension | | 120 | | | (24) | | | 37 | (30) | | 83 | (22) | | 0.185 | 28 | (31) | | 24 | (27) | | 0.098 |
| Distant site(s)/node(s) involved | | 53 | | | (10) | | | 7 | (6) | | 46 | (12) | | 0.228 | 6 | (7) | | 6 | (7) | | 0.000 |
| Unknown | | 19 | | | (4) | | | 9 | (7) | | 10 | (3) | | 0.215 | 4 | (4) | | 4 | (4) | | 0.000 |
| Barthel Index score | |  | | |  | | |  |  | |  |  | |  |  |  | |  |  | |  |
| 60-100 | | 453 | | | (90) | | | 108 | (87) | | 345 | (91) | | 0.110 | 79 | (88) | | 82 | (91) | | 0.109 |
| 40-59 | | 13 | | | (2) | | | 4 | (3) | | 9 | (2) | | 0.052 | 4 | (4) | | 1 | (1) | | 0.204 |
| 0-39 | | 39 | | | (8) | | | 12 | (10) | | 27 | (7) | | 0.094 | 7 | (8) | | 7 | (8) | | 0.000 |
| Overweight | | 165 | | | 33 | | | 45 | (36) | | 120 | (32) | | 0.102 | 28 | (31) | | 30 | (33) | | 0.047 |
| Current or past smoking | | 248 | | | (49) | | | 68 | (55) | | 180 | (47) | | 0.179 | 45 | (50) | | 47 | (52) | | 0.044 |
| Dyslipidemia | | 169 | | | (33) | | | 79 | (64) | | 90 | (24) | | 0.926 | 50 | (56) | | 53 | (59) | | 0.067 |
| Hypertension | | 223 | | | (44) | | | 84 | (68) | | 139 | (36) | | 0.669 | 55 | (61) | | 52 | (58) | | 0.068 |
| Diabetes mellitus | | 151 | | | (30) | | | 53 | (43) | | 98 | (26) | | 0.336 | 31 | (34) | | 37 | (41) | | 0.137 |
| Chronic kidney disease | | 29 | | | (6) | | | 7 | (6) | | 22 | (6) | | 0.004 | 6 | (7) | | 8 | (9) | | 0.083 |
| Congestive heart failure | | 86 | | | (17) | | | 35 | (28) | | 51 | (13) | | 0.359 | 20 | (22) | | 16 | (18) | | 0.111 |
| Atrial fibrillation | | 34 | | | (7) | | | 13 | (10) | | 21 | (6) | | 0.113 | 9 | (10) | | 7 | (8) | | 0.078 |
| β−blocker | | 108 | | | (21) | | | 41 | (33) | | 67 | (18) | | 0.389 | 28 | (31) | | 26 | (29) | | 0.048 |
| Statin | | 164 | | | (32) | | | 62 | (50) | | 102 | (27) | | 0.533 | 45 | (50) | | 46 | (51) | | 0.022 |
| ACE inhibitor | | 37 | | | (7) | | | 11 | (9) | | 26 | (7) | | 0.088 | 8 | (9) | | 6 | (7) | | 0.083 |
| ARB | | 143 | | | (28) | | | 34 | (27) | | 109 | (29) | | 0.002 | 29 | (32) | | 26 | (29) | | 0.072 |
| Oral anti-coagulants | | 53 | | | (10) | | | 14 | (11) | | 39 | (10) | | 0.048 | 11 | (12) | | 7 | (8) | | 0.148 |
| Acute coronary syndrome | | 106 | | | (21) | | | 42 | (34) | | 64 | (17) | | 0.356 | 25 | (28) | | 22 | (24) | | 0.076 |
| Days from cancer diagnosis to PCI/IHD admission, median (IQR) | | 253 | | | (59-852) | | | 385 | (153-656) | | 197 | (27-542) | | 0.349 | 371 | (136-636) | | 400 | (91-748) | | 0.095 |
| Chemo/radiation/hormonal therapy | | 307 | | | (61) | | | 74 | (60) | | 233 | (61) | | 0.045 | 53 | (59) | | 60 | (67) | | 0.161 |
| Surgery or endoscopic resection | | 107 | | | (21) | | | 23 | (19) | | 84 | (22) | | 0.068 | 20 | (22) | | 18 | (20) | | 0.054 |

Data are presented as n (%) unless otherwise indicated.

ACE, angiotensin-converting enzyme; ARB, angiotensin II receptor blocker; IHD, ischaemic heart disease; IQR, interquartile range; NA, not available; PCI, percutaneous coronary intervention; SD, standardized difference

**Supplemental Table S8 Baseline characteristics for gastric cancer as a subgroup analysis in the primary analysis**

|  |  | |  | |  | | Entire cohort | | | | | | | | Propensity score-matched sample | | | | | | | |
| --- | --- | --- | --- | --- | --- | --- | --- | --- | --- | --- | --- | --- | --- | --- | --- | --- | --- | --- | --- | --- | --- | --- |
|  | | All patients (n=1042) | | | | | | PCI+ (n=130) | | | PCI-/IHD+ (n=912) | | | SD^*^ | PCI+ (n=106) | | | PCI-/IHD+ (n=106) | | | SD^*^ | |
| Age, mean ± standard deviance | | 74 | | ± | | 7.9 | | 72 | ± | 7.6 | 74 | ± | 8.0 | 0.237 | 72 | ± | 7.6 | 73 | ± | 8.4 | 0.007 |  |
| Sex | |  | |  | |  | |  |  |  |  |  |  | 0.154 |  |  |  |  |  |  | 0.080 |  |
| Female | | 210 | | (20) | | | | 109 | (84) | | 189 | (21) | |  | 17 | (16) | | 14 | (13) | |  | |
| Male | | 832 | | (80) | | | | 21 | (16) | | 773 | (79) | |  | 89 | (84) | | 92 | (87) | |  | |
| Cancer stage | |  | |  | | | |  |  | |  |  | |  |  |  | |  |  | |  | |
| In situ | | 0 | | (0) | | | | 0 | (0) | | 0 | (0) | | NA | 0 | (0) | | 0 | (0) | | NA | |
| Localized | | 684 | | (66) | | | | 98 | (75) | | 586 | (64) | | 0.244 | 80 | (75) | | 84 | (79) | | 0.090 | |
| Regional to lymph nodes involved | | 129 | | (12) | | | | 11 | (8) | | 118 | (13) | | 0.145 | 7 | (7) | | 4 | (4) | | 0.128 | |
| Regional by direct extension | | 84 | | (8) | | | | 10 | (8) | | 74 | (8) | | 0.016 | 10 | (9) | | 8 | (7) | | 0.068 | |
| Distant site(s)/node(s) involved | | 126 | | (12) | | | | 9 | (7) | | 117 | (13) | | 0.199 | 7 | (7) | | 7 | (7) | | 0.000 | |
| Unknown | | 19 | | (2) | | | | 2 | (2) | | 17 | (2) | | 0.025 | 2 | (2) | | 3 | (3) | | 0.062 | |
| Barthel Index score | |  | |  | | | |  |  | |  |  | |  |  |  | |  |  | |  | |
| 60-100 | | 945 | | (91) | | | | 115 | (89) | | 830 | (91) | | 0.084 | 95 | (90) | | 90 | (85) | | 0.142 | |
| 40-59 | | 38 | | (3) | | | | 3 | (2) | | 35 | (4) | | 0.089 | 3 | (3) | | 4 | (4) | | 0.053 | |
| 0-39 | | 59 | | (6) | | | | 12 | (9) | | 47 | (5) | | 0.158 | 8 | (7) | | 12 | (11) | | 0.129 | |
| Overweight | | 241 | | (23) | | | | 29 | (22) | | 212 | (23) | | 0.014 | 24 | (23) | | 23 | (22) | | 0.023 | |
| Current or past smoking | | 575 | | (55) | | | | 81 | (62) | | 494 | (54) | | 0.186 | 68 | (64) | | 66 | (62) | | 0.039 | |
| Dyslipidemia | | 273 | | (26) | | | | 78 | (60) | | 195 | (21) | | 0.838 | 56 | (53) | | 57 | (54) | | 0.019 | |
| Hypertension | | 545 | | (52) | | | | 87 | (67) | | 458 | (50) | | 0.332 | 65 | (61) | | 65 | (61) | | 0.000 | |
| Diabetes mellitus | | 340 | | (33) | | | | 67 | (52) | | 273 | (30) | | 0.434 | 51 | (48) | | 51 | (48) | | 0.000 | |
| Chronic kidney disease | | 72 | | (7) | | | | 11 | (9) | | 61 | (7) | | 0.072 | 9 | (8) | | 9 | (8) | | 0.000 | |
| Congestive heart failure | | 167 | | (16) | | | | 43 | (33) | | 124 | (14) | | 0.449 | 29 | (27) | | 27 | (25) | | 0.043 | |
| Atrial fibrillation | | 109 | | (10) | | | | 11 | (8) | | 59 | (6) | | 0.080 | 9 | (8) | | 12 | (11) | | 0.094 | |
| β−blocker | | 247 | | (24) | | | | 49 | (38) | | 198 | (22) | | 0.367 | 34 | (32) | | 35 | (33) | | 0.020 | |
| Statin | | 290 | | (28) | | | | 68 | (52) | | 222 | (22) | | 0.617 | 53 | (50) | | 54 | (51) | | 0.019 | |
| ACE inhibitor | | 91 | | (9) | | | | 15 | (12) | | 76 | (8) | | 0.113 | 12 | (11) | | 10 | (9) | | 0.062 | |
| ARB | | 334 | | (32) | | | | 39 | (30) | | 295 | (32) | | 0.040 | 33 | (31) | | 34 | (32) | | 0.020 | |
| Oral anti-coagulants | | 109 | | (10) | | | | 12 | (9) | | 97 | (11) | | 0.042 | 10 | (9) | | 14 | (13) | | 0.119 | |
| Acute coronary syndrome | | 179 | | (17) | | | | 34 | (26) | | 145 | (16) | | 0.262 | 23 | (22) | | 26 | (25) | | 0.067 | |
| Days from cancer diagnosis to PCI/IHD admission, median (IQR) | | 71 | | (37-306) | | | | 240 | (97-645) | | 66 | (36-237) | | 0.591 | 200 | (68-559) | | 95 | (45-514) | | 0.067 | |
| Chemo/radiation/hormonal therapy | | 237 | | (23) | | | | 18 | (14) | | 219 | (24) | | 0.255 | 14 | (13) | | 14 | (13) | | 0.000 | |
| Surgery or endoscopic resection | | 810 | | (78) | | | | 108 | (83) | | 702 | (77) | | 0.146 | 86 | (81) | | 86 | (81) | | 0.000 | |

Data are presented as n (%) unless otherwise indicated.

ACE, angiotensin-converting enzyme; ARB, angiotensin II receptor blocker; IHD, ischaemic heart disease; IQR, interquartile range; NA, not available; PCI, percutaneous coronary intervention; SD, standardized differene

**Supplemental Table S9** **Multivariable analysis of mortality with propensity score matching and immortal time bias adjustment (secondary analysis)**

|  | **HR** | **[95% CI]** | **P-value** |
| --- | --- | --- | --- |
| PCI | 2.03 | [1.35–3.07] | 0.001 |
| Age | 1.06 | [1.03–1.09] | <0.001 |
| Sex |  |  |  |
| Male | Ref |  |  |
| Female | 0.38 | [0.16–0.90] | 0.027 |
| Cancer type |  |  |  |
| Colorectal cancer | Ref |  |  |
| Lung cancer | 2.77 | [1.63–4.71] | <0.001 |
| Prostate cancer | 0.39 | [0.16–0.95] | 0.038 |
| Gastric cancer | 2.17 | [1.23–3.83] | 0.007 |
| Cancer stage |  |  |  |
| In situ | Ref |  |  |
| Localised | 2.62 | [0.34–20.14] | 0.353 |
| Regional to lymph nodes involved | 9.20 | [1.20–70.65] | 0.033 |
| Regional by direct extension | 8.41 | [1.05–67.07] | 0.045 |
| Distant site(s)/node(s) involved | 20.61 | [2.70–157.15] | 0.004 |
| Unknown | 28.62 | [3.28–249.66] | 0.002 |
| Barthel Index score |  |  |  |
| 60–100 | Ref |  |  |
| 40–59 | 2.60 | [0.34–19.67] | 0.354 |
| 0–39 | 4.51 | [2.35–8.66] | <0.001 |

CI, confidence interval; HR, hazard ratio; PCI, percutaneous coronary intervention; Ref, reference


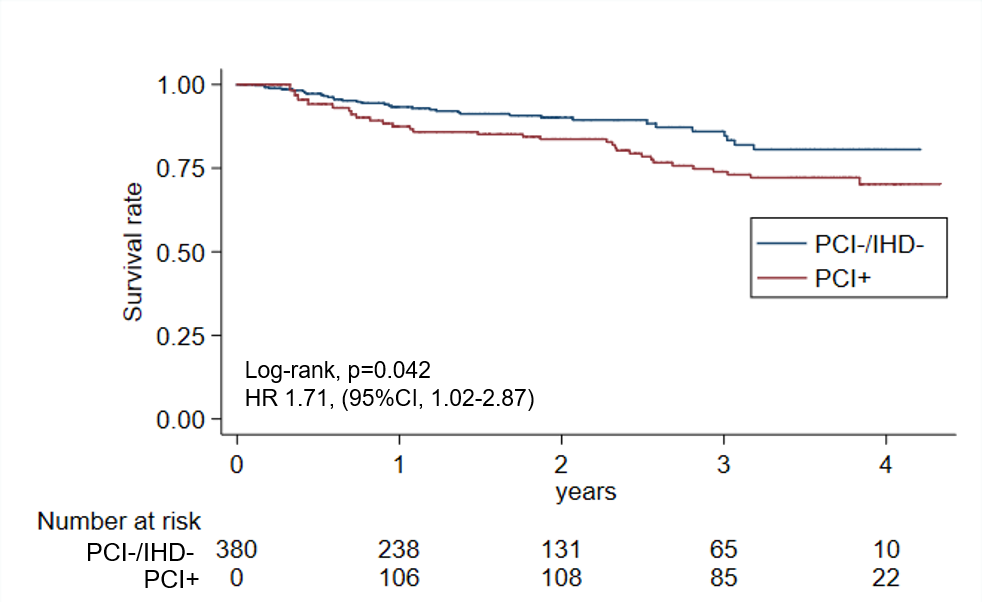


**Supplemental Figure S4 Kaplan-Meier analysis of all-cause mortality for PCI+ group patients without acute coronary syndrome and PCI-/IHD- group patients as a sensitivity analysis for the secondary analysis**

Data of the PCI+ group patients without acute coronary syndrome and PCI-/IHD- group patients were adjusted for background factors used in the prior analyses. After propensity score matching, immortal time bias was adjusted by counting the periods from cancer diagnosis to PCI admission for the PCI+ group and PCI-/IHD- group since during this period, the PCI+ group had no patients at risk. The origin of the Kaplan-Meier analysis was at the cancer diagnosis for both groups; however, the PCI+ group was allowed to contribute risk to the PCI-/IHD- group before the PCI.

HR, hazard ratio; CI, confidence interval; IHD, ischaemic heart disease; PCI, percutaneous coronary intervention

**Supplemental Table S10** **Baseline characteristics for the PCI+ group wihout acute coronary syndrome and PCI-/IHD- group**

|  |  |  |  | Entire cohort | | | | | | | Propensity score-matched sample | | | | | | |
| --- | --- | --- | --- | --- | --- | --- | --- | --- | --- | --- | --- | --- | --- | --- | --- | --- | --- |
|  | All patients (n=27593) | | | PCI+ (n=201) | | | PCI-/IHD+ (n=27392) | | | SD^*^ | PCI+ (n=190) | | | PCI-/IHD+ (n=190) | | | SD^*^ |
| Age, mean ± standard deviance | 70 | ± | 10.2 | 73 | ± | 6.8 | 70 | ± | #### |  | 73 | ± | 6.8 | 74 | ± | 6.5 | 0.061 |
| Sex |  |  |  |  |  |  |  |  |  | 0.480 |  |  |  |  |  |  | 0.031 |
| Female | 8550 | (31) | | 24 | (12) | | 8526 | (31) | |  | 24 | (13) | | 26 | (14) | |  |
| Male | 19043 | (69) | | 177 | (88) | | 18866 | (69) | |  | 166 | (87) | | 164 | (86) | |  |
| Cancer type |  |  |  |  |  | |  |  | |  |  |  | |  |  | |  |
| Colorectal cancer | 9772 | (35) | | 60 | (30) | | 9712 | (35) | | 0.120 | 57 | (30) | | 63 | (33) | | 0.068 |
| Lung cancer | 5978 | (22) | | 49 | (24) | | 5929 | (22) | | 0.065 | 47 | (25) | | 42 | (22) | | 0.062 |
| Prostate cancer | 4303 | (16) | | 41 | (21) | | 4262 | (16) | | 0.126 | 36 | (19) | | 35 | (19) | | 0.014 |
| Gastric cancer | 7540 | (27) | | 51 | (25) | | 7489 | (27) | | 0.045 | 50 | (26) | | 50 | (26) | | 0.000 |
| Cancer stage |  |  | |  |  | |  |  | |  |  |  | |  |  | |  |
| In situ | 2561 | (9) | | 13 | (7) | | 2548 | (9) | | 0.105 | 11 | (6) | | 11 | (6) | | 0.000 |
| Localized | 12707 | (46) | | 117 | (58) | | 12590 | (46) | | 0.247 | 110 | (58) | | 115 | (60) | | 0.054 |
| Regional to lymph nodes involved | 2910 | (11) | | 25 | (12) | | 2885 | (11) | | 0.060 | 25 | (13) | | 25 | (13) | | 0.000 |
| Regional by direct extension | 2810 | (10) | | 28 | (14) | | 2782 | (10) | | 0.116 | 26 | (14) | | 22 | (12) | | 0.063 |
| Distant site(s)/node(s) involved | 5837 | (21) | | 16 | (8) | | 5821 | (21) | | 0.383 | 16 | (8) | | 17 | (9) | | 0.019 |
| Unknown | 768 | (3) | | 2 | (1) | | 766 | (3) | | 0.132 | 2 | (1) | | 0 | (0) | | 0.146 |
| Barthel Index score |  |  | |  |  | |  |  | |  |  |  | |  |  |  |  |
| 60-100 | 25628 | (93) | | 194 | (97) | | 25434 | (93) | | 0.164 | 184 | (97) | | 184 | (97) | | 0.000 |
| 40-59 | 693 | (2) | | 2 | (1) | | 691 | (2) | | 0.116 | 2 | (1) | | 2 | (1) | | 0.000 |
| 0-39 | 1272 | (5) | | 5 | (2) | | 1267 | (5) | | 0.116 | 4 | (2) | | 4 | (2) | | 0.000 |
| Overweight | 6094 | (22) | | 63 | (31) | | 6031 | (22) | | 0.212 | 55 | (29) | | 58 | (31) | | 0.035 |
| Current or past smoking | 14092 | (51) | | 122 | (61) | | 13970 | (51) | | 0.196 | 117 | (62) | | 112 | (59) | | 0.054 |
| Dyslipidemia | 559 | (2) | | 13 | (6) | | 546 | (2) | | 0.224 | 8 | (4) | | 7 | (4) | | 0.027 |
| Hypertension | 1385 | (5) | | 21 | (10) | | 1364 | (5) | | 0.206 | 13 | (7) | | 11 | (6) | | 0.043 |
| Diabetes mellitus | 1128 | (4) | | 25 | (12) | | 1103 | (4) | | 0.310 | 17 | (9) | | 12 | (6) | | 0.099 |
| Chronic kidney disease | 216 | (1) | | 9 | (4) | | 207 | (1) | | 0.235 | 3 | (2) | | 7 | (4) | | 0.132 |
| Congestive heart failure | 249 | (1) | | 10 | (5) | | 239 | (1) | | 0.245 | 3 | (2) | | 4 | (2) | | 0.039 |
| Atrial fibrillation | 308 | (1) | | 0 | (0) | | 308 | (1) | | 0.151 | 0 | (0) | | 0 | (0) | | NA |
| β−blocker | 556 | (2) | | 24 | (12) | | 532 | (2) | | 0.401 | 14 | (7) | | 12 | (6) | | 0.042 |
| Statin | 1237 | (4) | | 32 | (16) | | 1205 | (4) | | 0.388 | 25 | (13) | | 21 | (11) | | 0.065 |
| ACE inhibitor | 296 | (1) | | 4 | (2) | | 29 | (1) | | 0.058 | 3 | (2) | | 1 | (1) | | 0.032 |
| ARB | 1741 | (6) | | 34 | (17) | | 1707 | (6) | | 0.329 | 24 | (13) | | 22 | (12) | | 0.032 |
| Oral anti-coagulants | 244 | (1) | | 5 | (2) | | 239 | (1) | | 0.050 | 2 | (1) | | 4 | (2) | | 0.032 |
| Acute coronary syndrome | - | - | | - | - | | - | - | | NA | 0 | (0) | | 0 | (0) | | NA |
| Days from cancer diagnosis to PCI/IHD admission, median (IQR) | - | - | | 238 | (95-562) | | - | - | | NA | 236 | (95-570) | | - | - | | NA |
| Chemo/radiation/hormonal therapy | 10189 | (37) | | 66 | (33) | | 10123 | (37) | | 0.127 | 60 | (32) | | 57 | (30) | | 0.049 |
| Surgery or endoscopic resection | 16578 | (60) | | 143 | (71) | | 16435 | (60) | | 0.343 | 139 | (73) | | 140 | (74) | | 0.016 |

Data are presented as n (%) unless otherwise indicated.

ACE, angiotensin-converting enzyme; ARB, angiotensin II receptor blocker; IHD, ischaemic heart disease; IQR, interquartile range; NA, not available; PCI, percutaneous coronary intervention; SD, standardized difference
